# Supplementary material for: Fibroblastic reticular cell-derived lysophosphatidic acid regulates confined intranodal T-cell motility
Source: eLife. 2016 Feb 2;5:e10561. doi: 10.7554/eLife.10561 (PMC4755752; doi:10.7554/eLife.10561)
Supplement: Supplementary file 1. — These primers were designed using Primer3 software (Sourceforge) and used for SYBR green-based real time-PCR analysis. DOI: http://dx.doi.org/10.7554/eLife.10561.021 [file elife-10561-supp1.docx]

| **Gene** | **Forward** | **Reverse** |
| --- | --- | --- |
| *Enpp2* | TTGTCCGCCCTCCGTTAATC | ACAGGACCGCAGTTTCTCAA |
| *Cxcl13* | TCGTGCCAAATGGTTACAAA | ACAAGGATGTGGGTTGGGTA |
| *Ccl19* | CTGCCTCAGATTATCTGCCAT | AGGTAGCGGAAGGCTTTCAC |
| *Ccl21* | AAGGCAGTGATGGAGGGG | CGGGGTAAGAACAGGATTG |
| *Icam1* | CACGTGCTGTATGGTCCTCG | TAGGAGATGGGTTCCCCCAG |
| *Glycam1* | GAGAATCAAGAGGCCCAGGAT | TGGGTCTTGTGGTCTCTTCCA |
| *Lpar1* | CCTCTTCATCGCCCCAAACT | TTCATGGCTGTGAACTGGGG |
| *Lpar2* | CATTCTGGGGGCATTTGTGG | TTGACCAGTGAGTTGGCCTC |
| *Lpar3* | GTCTTAGGCGCCTTCGTGG | TTGCACGTTACACTGCTTGC |
| *Lpar4* | GCGAGTTGCCAGTTTACACG | TTGAGTGCCCAAGAAAGAGTGT |
| *Lpar5* | CAAGAAGGTCTCCACTGCTGA | GTGGTAGCCTGGTGGCAATA |
| *Lpar6* | CTGTAAGCTGCACTGCCTGA | ATGCTGAACATGCACCCGTA |
| *Gapdh* | CCTCGTCCCGTAGACAAAATG | TCTCCACTTTGCACTGCAA |

**Supplementary File 2. Primer sequences for quantitative PCR analysis.**

These primers were designed using Primer3 software (Sourceforge) and used for SYBR^®^ green-based real time-PCR analysis.
